# The Pharmaceutical Industry in 2019. An Analysis of FDA Drug Approvals from the Perspective of Molecules

**DOI:** 10.3390/molecules25030745

**Published:** 2020-02-09

**Authors:** Beatriz G. de la Torre, Fernando Albericio

**Affiliations:** 1 KRISP, College of Health Sciences, University of KwaZulu-Natal, Durban 4001, South Africa; 2 School of Chemistry and Physics, University of KwaZulu-Natal, Durban 4001, South Africa; 3 CIBER-BBN, Networking Centre on Bioengineering, Biomaterials and Nanomedicine, Department of Organic Chemistry, University of Barcelona, 08028 Barcelona, Spain

**Keywords:** antibodies, antibody drug conjugate, API, biologics, chemical entities, drug discovery, fluorine-based drugs, natural products, oligonucleotides, peptides, pyrazoles, TIDES, small molecules

## Abstract

During 2019, the US Food and Drug Administration (FDA) approved 48 new drugs (38 New Chemical Entities and 10 Biologics). Although this figure is slightly lower than that registered in 2018 (59 divided between 42 New Chemical Entities and 17 Biologics), a year that broke a record with respect to new drugs approved by this agency, it builds on the trend initiated in 2017, when 46 drugs were approved. Of note, three antibody drug conjugates, three peptides, and two oligonucleotides were approved in 2019. This report analyzes the 48 new drugs of the class of 2019 from a strictly chemical perspective. The classification, which was carried out on the basis of chemical structure, includes the following: Biologics (antibody drug conjugates, antibodies, and proteins); TIDES (peptide and oligonucleotides); drug combinations; natural products; and small molecules.

## 1. Analysis

**Figure 1:**
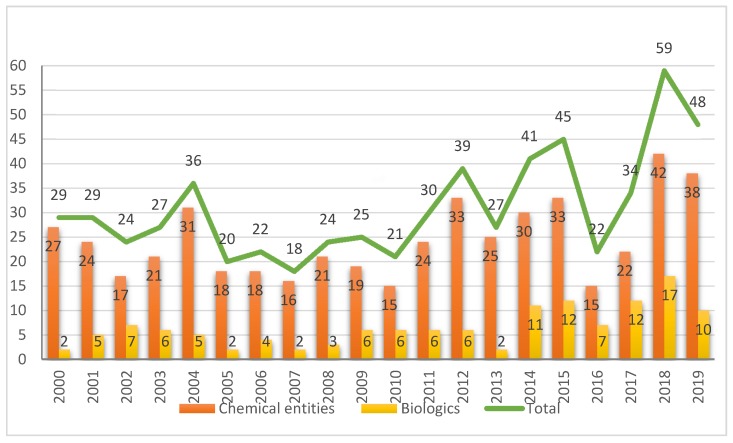
New chemical entities and biologics approved by the FDA in the last two decades [1,6].

In terms of the number of drugs approved by the Food and Drug Administration (FDA), 2019 (also referred to as “this year” herein) saw with the approval of 48 new drugs, a figure that consolidates the trend initiated in 2017, when 46 drugs were approved, followed by a record-breaking 59 approvals in 2018 [1]. The 48 new drugs of 2019 are divided between 10 Biologics (12 and 17 in 2017 and 2018, respectively) and 38 New Chemical Entities (NCEs) (34 and 42 in 2017 and 2018, respectively) Figure 1 [1]. In this same forum [2] last year, and in response to the large number of drugs accepted in 2018, we asked the following question: *will this increasing trend of both kinds of drug continue in the coming years?* We and other analysts were cautious in this regard [2,3,4], since the approval of a new drug by the corresponding agencies involves many variables that are difficult to predict. After seeing the results of 2019, our analyses are guardedly optimistic in the expectation that this trend will be maintained in the near future [5,6]. In this respect, it is important to take into consideration that the pharmaceutical industry heads the world ranking in total Research and Development (R&D) investment and it is one of the few sectors still growing.

While the number of NCEs approved in 2019 is in line with the expectations based on previous years, the number of Biologics could be considered somewhat disappointing after the figures registered in 2017 and 2018 [1]. However, the approval of three antibody drug conjugates (ADCs) could be considered a breakthrough and something awaited by analysts for several years [7,8,9]. The approvals of ADCs granted in 2019 account for almost 50% (3 vs. 7) of all ADCs approved by the FDA to date.

Overall, Biologics are clearly consolidated in the drug arena, accounting for more than 25% (58 out of 220) of all drugs approved in the last five years (2015–2019). In parallel, in 2019, the Center for Biologics Evaluation and Research (CBER) has added 10 new approvals, including three vaccines and one gene therapy [10]. This number clearly exceeds those of the previous years (6 and 3 in 2017 and 2018, respectively) and paves the way for their further application for these kinds of treatments [10].

## 2. Discussion

Ten Biologics were approved in 2019 as shown in Table 1, of which three were ADCs, five monoclonal antibodies (mAbs), and two proteins—one a fusion protein and one a neurotoxin (Table 1).

Although mAbs continue to be an important class of Biologics and even of drugs, 2019 registered a slight decrease in the number of mAbs approved by the FDA. The number of mAb authorized by this regulatory agency has experienced annual increases (7, 9, and 11 for 2016, 2017, and 2018, respectively); however, the five approved in 2019, which still account for 10% of total drugs and the 50% of Biologics authorized, is the lowest number in recent years. Of note, one of these mAb, Caplacizumab (Cablivi^TM^), is a nanobody that contains a single-domain antibody fragment. This is the first nanobody to make it to the market. Importantly, the medicinal targets of mAb continue to be broad, as shown in 2019: eye and blood diseases, osteoporosis and psoriasis (Table 1).

In this same report in 2018 [2], we drew attention to a forecast made by analysts, namely that that more ADCs would be approved by the FDA in the coming years. In 2019, this prediction became a reality, with the FDA approval three ADCs. With these ADCs approved this year, the FDA has authorized seven ADCs to date, all of which are indicated for the treatment of cancer.

**Figure 2:**
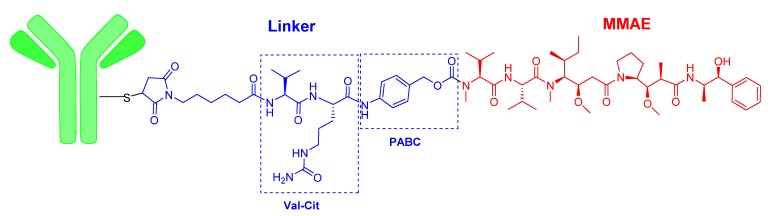
Structure of enfortumab vedotin and polatuzumab vedotin.

Two of this year’s ADCs, namely enfortumab vedotin-ejfv (Padcev^TM^) and polatuzumab vedotin-piiq (Polivy^TM^), share the same structure, differing only in the mAb (Figure 2). The payload drug of both agents is monomethyl auristatin E (MMAE), which is a peptide derived from the marine mollusc dolastatin that is too toxic to be administered as a drug itself. MMAE is also the drug contained in brentuximab vedotin (Adcetris^TM^), which was approved by the FDA in 2011. In the three ADCs, the cleavable linker is the dipeptide Val-Cit, which contains the auto-inmolative moiety p-aminobenzyl alcohol carbamate (PABC) at the C-terminal. Val-Cit is cleavable by the protease cathepsin, releasing the PABC bound to the MMAE. In turn, the PABC moiety spontaneously releases the MMAE. At the N-terminal of Val-Cit, the mAb is bound through the thiol of a Cys via a Michael addition to 6-maleimidohexanoyl linked to the N-amino of the Val (Figure 2). Enfortumab vedotin-ejfv is indicated for cancers expressing Nectin-4, such urothelial cancers.

**Figure 3:**
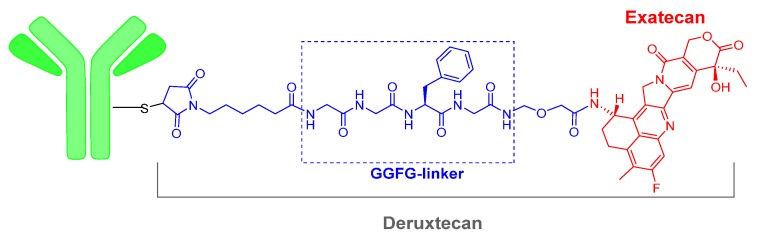
Structure of Fam-trastuzumab deruxtecan.

The third ADC of 2019, Fam-trastuzumab deruxtecan-nxki (Enhertu^TM^) is the same mAb contained in another ADC, trastuzumab emtansine (Kadcyla^TM^), which was approved in 2013. This mAb is also commercialized alone as Herceptin^TM^. The payload drug on Enhertu^TM^ is the topoisomerase I inhibitor deruxtecan, which is a derivative of exatecan. As in the two other ADCs of this year, the mAb is linked to the payload, deruxtecan, through Cys residues incorporated into a 6-maleimidohexanoyl moiety (Figure 3). Deruxtecan also contains the tetrapeptide Gly–Gly–Phe–Gly (GGFG), which is a protease cleavable linker releasing an analogue of exatecan. Fam-trastuzumab deruxtecan-nxki is indicated for metastatic breast cancer.

In addition to the five ADCs mentioned above, the FDA also approved cemtuzumab ozogamicin (Mylotarg^TM^) in 2001. However, authorization was withdrawn in 2010; the drug was later re-introduced in 2017. Inotuzumab ozogamicin (Besponsa^TM^) was also approved in 2017. Furthermore, it is important to highlight that in 2018, two drugs based on the same idea but with a different chemical construction to that of the ADCs were approved. Moxetumomab pasudotox (Lumoxiti^TM^) is a recombinant immunotoxin formed by an antibody covalently bound to a fragment of Pseudomonas exotoxin-A, and tagraxofusp-erzs (Elzonris^TM^) is an interleukin 3-based fusion protein containing the diphtheria toxin. All these approvals reinforce the notion of the benefits of constructing chimeras of mAb/protein and toxic moieties.

Prabotulinumtoxin (botulinum toxin, Jeuveau^TM^) deserves comment. This toxin, which is probably the most lethal toxin known, had already been used extensively in cosmetics to reduce facial wrinkles and it had several *pros* and *cons.* One advantage was evident cosmetic effects; however, inappropriate administration can lead to the paralysis of unintended muscles. Prabotulinumtoxin shows an improved purity that contributed to its improved safety profile, therefore its approval by the FDA is expected to increase the safety of its use as a cosmetic.

Although TIDES (oligonucleo- and pep-TIDES) are synthesized chemically, they fall among Biologics and the so-called small molecules. For TIDES, 2019 was another excellent year. In this regard, in addition to the two ADCs based on MMAE, a peptide of marine origin, three more peptides and two oligonucleotides were received the green light from the FDA. This number indicates that more than 10% of the total drugs approved by this agency in 2019 were TIDES.

**Figure 4:**
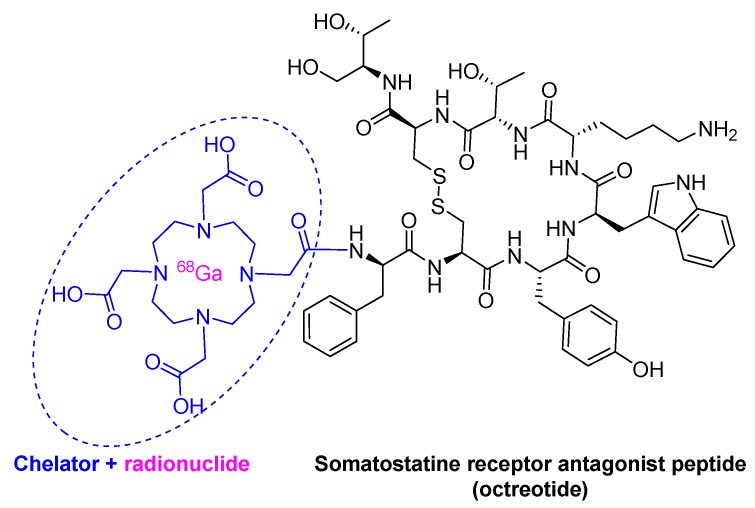
Structure of Ga 68 dodecanetetraacetic acid-Tyr3-octeotride (DOTA-TOC).

Gallium Ga 68 DOTA-TOC, composed by the cyclic octapeptide (Tyr^3^-octeotride, TOC) terminated at the C-terminal with threoninol and at the *N*-terminal with a dodecanetetraacetic acid (DOTA) chelator, to which Ga-68 is bound, received approval in 2019 (Figure 4). Ga 68 DOTA-TATE is used in diagnostics to measure the density and whole-body biodistribution of tumor somatostatin receptors (SSRs) via positron emission tomography (PET) imaging. In 2018, Lutetium Lu 177 DOTA-TATE (Lutathera^TM^), which is build up in turn to octeotrate or octreotide acid (Thr as C-terminal) was approved for peptide receptor radionuclide therapy and PET imaging. Lu 177 DOTA-TATE is considered a theragnostic drug, therapy, and diagnosis, for neuroendocrine tumors.

**Figure 5:**
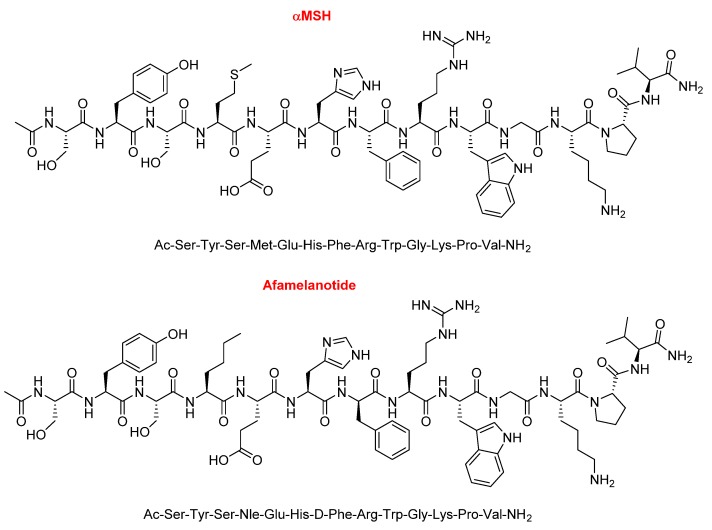
Structure of α-melanocyte-stimulating hormone (αMSH) vs. afamelanotide.

Afamelanotide (Scenesse^TM^), first known as melanotan I, is a 13mer lineal peptide with a C-terminal amide and acetylated N-terminal (Figure 5). It is formed by proteinogenic amino acids, except two, a norleucine (Nle) and a D-phenylalanine. It is a potent α-melanocyte-stimulating hormone (αMSH) analogue (Figure 5). It promotes the production of eumelanin in the skin and is used to prevent skin damage and the concomitant pain experienced by people with erythropoietic protoporphyria when exposed to the sun. Due to its short half-life of 30 min, afamelanotide is administered in subcutaneous implant form and it lasts for two months.

**Figure 6:**
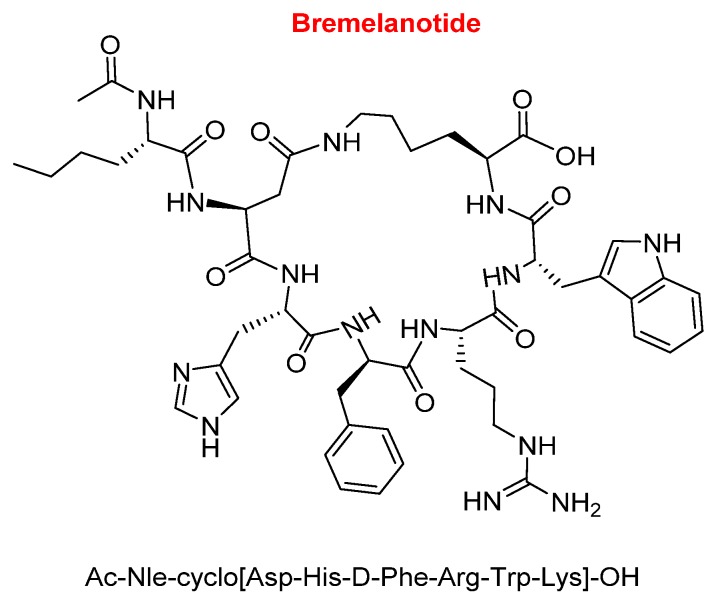
Structure of bremelanotide.

Bremelanotide (Vyleesi^TM^) is a hepta side-chain to side-chain homodetic cyclic peptide (formed only by amide bonds), where the bridge is between the side-chain of the Lys at the C-terminal and that of the Asp at position 2. While the Lys residue is in the form of an acid, the exocyclic part is formed by an acetylated Nle (Figure 6). Bremelanotide is indicated for the treatment of hypoactive sexual desire disorder in premenopausal women with no further problems (psychiatric and/or of the relationship).

**Figure 7:**
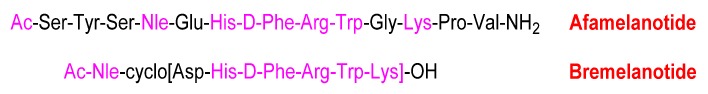
Comparison of the sequences of afamelanotide and bremelanotide (in pink, the common part).

Like the previous peptide, bremelanotide is also an analogue of α-MSH. Comparison of the sequences of bremelanotide and afamelanotide reveals that the former has a shorter sequence and lacks the following parts: The tripetide Ser–Tyr–Ser at the N-terminal, the dipeptide Pro-Val at the C-terminal, and the Gly (Figure 7). Furthermore, the Glu of bremelanotide is substituted by Asp, which forms the bridge with the ε-amino of the Lys.

**Figure 8:**
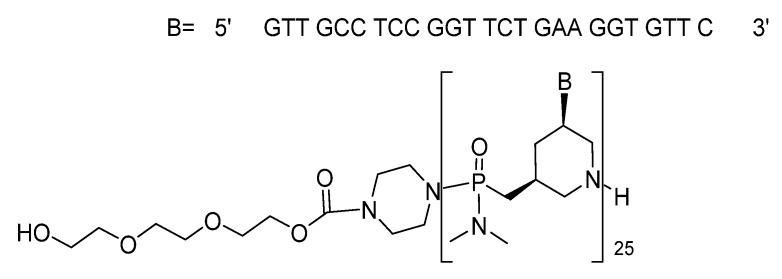
Structure of Golodirseen.

With regards to the oligonucleotide class, following the trends of 2016 and 2018, when three and two oligos were approved by the FDA, respectively, in 2019 two oligos were approved—one phosphorodiamidate morpholino oligomer (PMO) and one a double-stranded short interfering RNA (siRNA). Golodirsen (Vyondys 53^TM^) is used for the treatment of Duchenne muscular dystrophy (DMD) with genetic mutations subject to skipping exon 53 of the dystrophin gene. It is an antisense 25 mer PMO ending at 5’ with a short polyethyleneglycol chain (Figure 8). Golodirsen was first refused by FDA (August 2019), and after the appeal by Sarepta Therapeutics, it was authorized in December 2019. Another PMO, eterlipsen (Exondys 51^TM^), from the same company, was approved in 2016.

**Figure 9:**
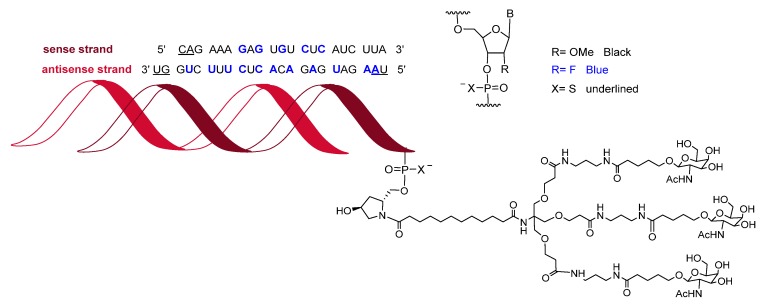
Structure of givosiran.

Givosiran (Givlaari^TM^) is used for the treatment of adults with acute hepatic porphyria, a genetic disorder that causes the buildup of toxic porphyrin molecules, which are formed during the production of heme, a compound that is essential for blood-oxygen binding. Givosiran is a double-stranded siRNA. Both strands contain in total 16 2’-F-ribonucleosides units and six thiphosphate linkages. The F improved the stability of the double-strand. Givosiran was developed by Alnylam, the same company that last year saw the approval of patisiran (Onpattro^TM^). While patisiran is encapsulated in a lipid nanoparticle, givosiran is built through a proprietary Enhanced Stabilization Chemistry (ESC), where in addition of F- and methoxy-ribonucleosides, the short dendrimer bearing N-acetylgalactosamine (GalNAc) conjugated to the sense strand, there are six thiophosphate linkages at the other ends (Figure 9). Furthermore, the presence of GalNAc mediates its binding and internalization by hepatocytes.

These seven oligonucleotide-based drugs approved between 2016 and 2019 are a clear indication of the potential of this class of compounds and they are expected to fuel the development of more drugs belonging to this class. Furthermore, these drugs should now bring about a return on the investment made by several pharmaceutical industries over the last thirty years.

As in recent years, where drugs containing more than one API were approved, 2019 has witnessed the authorization of two combination drugs: Trikafta^TM^ to treat cystic fibrosis (CF) and Recarbrio^TM^ as an antibiotic. Both are triple combination drugs.

**Figure 10:**
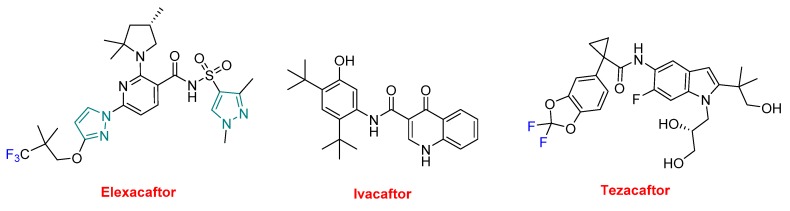
Structure of Trikafta^TM^, a drug combination (in green the structure of pyrazole, in blue the fluorine).

Trikafta^TM^ is formed by elexacaftor, ivacaftor (Kalydeco^TM^), and tezacaftor (Figure 10). Ivacaftor was approved alone in 2012, in combination with lumacaftor under the trade name of Orkambi^TM^ in 2015, and with tezacaftor —also present in Trikafta^TM^—under the name of Symdeko^TM^ in 2018 for several variations of CF. In this regard, ivacaftor is present in four APIs. A common characteristic of all these drugs is the yearly cost of the treatment, which reaches a 6-digit figure.

**Figure 11:**
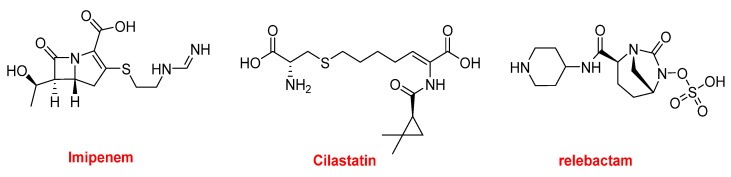
Structure of Recarbrio^TM^, a combination drug.

Recarbrio^TM^ is formed by imipenem, cilastatin, and relebactam (Figure 11). Imipenem (Primaxin^TM^ among others) is a β-lactam antibiotic that was approved in 1985. It belongs to the carbapenem family and is derived from the natural product thienamycin. It has a broad spectrum of activity against Gram-positive and Gram-negative bacteria. Cilastatin is a derivative of the amino acid cysteine and it inhibits the human enzyme dehydropeptidase, which is responsible for degrading the imipenem. Finally, relebactam is a beta-lactamase inhibitor. Recarbrio^TM^ is specifically recommended for complicated urinary tract and intra-abdominal infections.

**Figure 12:**
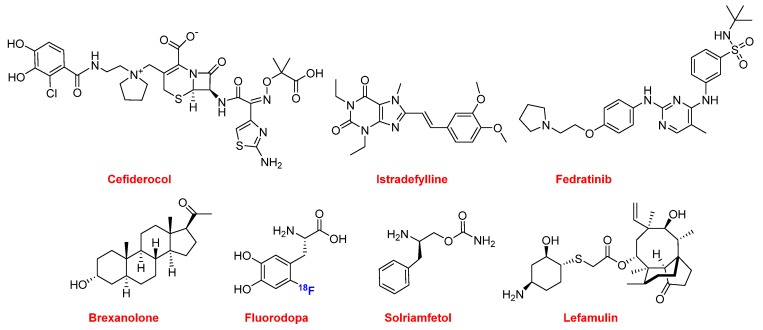
Structure of the natural product-based drugs (in blue the fluorine).

2019 has once again been a good year for drugs inspired on natural products. In addition to imipenem and cilastatin, seven more drugs from this source were approved, thereby highlighting the importance of natural products in the drug discovery process (Figure 12).

Cefiderocol (Fetroja^TM^) is from the cephalosporin family and is indicated for the treatment of urinary tract infection caused mainly by multidrug-resistant Gram-negative bacteria.

Two drugs belong to the family of the bases found in nucleic acids. Istradefylline (Nourianz^TM^) belongs to the purines and it is administered as an add-on to levodopa/carbidopa patients with Parkinson’s disease experiencing “off” episodes. Fedratinib (Inrebic^TM^) is a pyrimidine and a semi-selective inhibitor of Janus kinase 2 (JAK-2). It is indicated for the treatment of myeloproliferative diseases.

In the steroid family, allopregnanolone or brexanolone (Zulresso^TM^) was approved this year for the treatment of postpartum depression.

The fluorine-18 isotopologue of L-DOPA, fluorodopa (FDOPA^TM^), was approved as a radiotracer in PET for the visualization of nerve cells in patients with symptoms of Parkinson’s disease. Also derived from the amino acid phenylalanine, Solriamfetol (Sunosi^TM^) is indicated for symptoms associated with sleepiness such as narcolepsy and sleep apnea.

Finally, in this natural product chapter, lefamulin (Xenleta^TM^) is an antibiotic to treat community-acquired bacterial pneumonia. Lefamulin belongs to the family of pleuromutilin, which is derived from the fungus *Clitopilus passeckerianus.* Although pleuromutilin antibiotics have been widely used in veterinary medicine, lefamulin is the first to be used for systemic treatment of bacterial infections in humans.

In addition to givosiran, elexacaftor, and tezacaftor (both part of Trikafta^TM^), fluorodopa, and entrectinib (see below in the pyrazole section), another 10 drugs contain fluorine (in blue in the figures). This implies that more than a quarter (14 out of 48) of all drugs approved by the FDA during 2019 contain this atom. Considering only the NCEs, this proportion increases to slightly more than a third (13 vs. 38). This observation clearly emphasizes the significant impact of fluorine in the drug arena.

**Figure 13:**
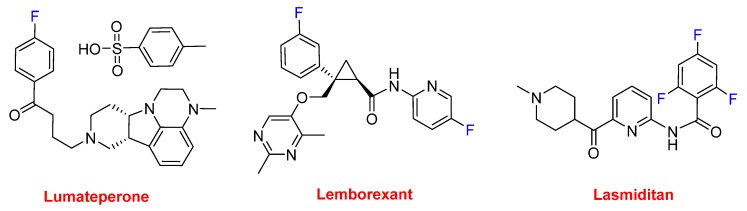
Structure of drugs containing fluoroaryl moieties.

Lumateperone tosylate (Caplyta^TM^), which contains a fluorophenyl moiety, belongs to the butyrophenone family and is indicated for the treatment of schizophrenia and is currently in development for bipolar depression and other neurological indications. Lemborexant (Dayvigo^TM^) contains a fluorophenyl and a fluoropyridinyl moiety and is indicated for the treatment of insomnia. Lasmiditan (Reyvow^TM^) contains a trifluorobenzoyl moiety and is recommended for the acute treatment of migraine (Figure 13).

**Figure 14:**
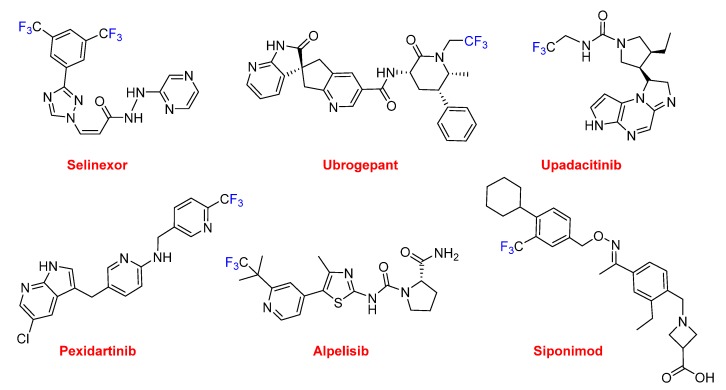
Structures of drugs containing trifluoromethyl groups.

Selinexor (Xpovio^TM^), which contains two trifluoromethyl groups, is an anti-anticancer drug (multiple myeloma). It is the first in class with a novel mechanism of action based on blocking the transport of several proteins involved in cancer cell growth from the cell nucleus to the cytoplasm, thereby ultimately arresting the cell cycle and leading to apoptosis. There are six drugs with just one trifluoromethyl group. Ubrogepant (Ubrelvy^TM^) is also indicated for the acute treatment of migraine; however, it has a different mechanism of action to lasmiditan. Ubrogepant is the first drug in class of oral calcitonin gene-related peptide receptor antagonists. Upadacitinib (Rinvoq^TM^) is approved for the treatment of rheumatoid arthritis. Pexidartinib (Turalio^TM^) is indicated for the treatment of giant-cell tumor of the tendon sheath (GC-TS). Alpelisib (Piqray^TM^) is recommended to treat certain types of breast cancer. Siponimod (Mayzent^TM^) is used for relapsing multiple sclerosis (MS) and is a selective sphingosine-1-phosphate receptor modulator (Figure 14).

**Figure 15:**
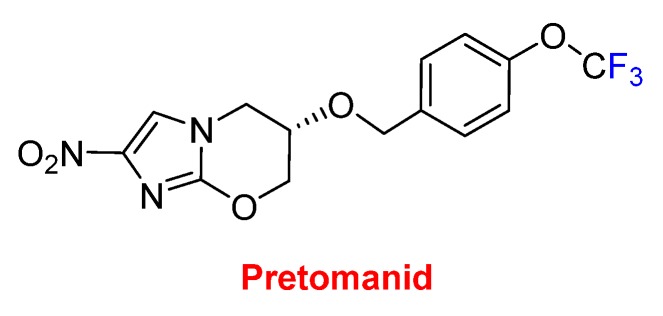
Structure of pretomanid.

These six drugs contain either trifluoromethyl aryl or alkyl moieties. The last one, pretomanid, contains a trifluoromethoxy moiety and is used for the treatment of multi-drug-resistant tuberculosis (Figure 15). It is administered in a combination regimen with bedaquiline and linezolid. Furthermore, pretomanid has an interesting history. The “preto” prefix honors the administrative capital of South Africa, Pretoria, which is the home of the non-profit TB Alliance. Pretomanid is the first drug to be developed and registered by a not-for-profit organization and the third new anti-TB drug approved for use by the FDA in more than 40 years. It is a member of the family of the nitroimidazooxazines.

**Figure 16:**
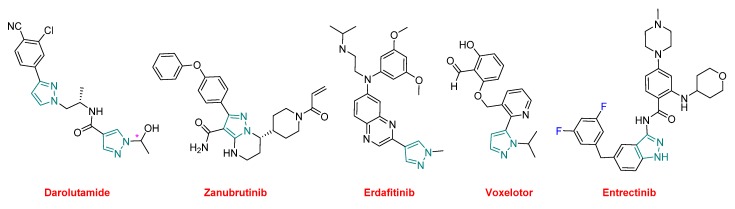
Structure of drugs, containing pyrazole/indazole moieties (* denotes a chiral center).

All the small molecules in 2019, except five, contain nitrogen heterocycles, thereby indicating a certain supremacy of this kind of chemistry and accounting for almost 85% (27 vs. 32) of this class of drugs. Of all these APIs approved this year, six contain pyrazole, or its related indazole, moieties (both are indicated in green in the figures) (Figure 16). Elexacaftor, which is part of Trikafta^TM^, contains two pyrazole rings. Darolutamide (Nubeqa^TM^), which also contains two of these heterocycles, was approved for the treatment of non-metastatic castration-resistant prostate cancer (nmCRPC). Zanubrutinib (Brukinsa^TM^) is a Bruton’s tyrosine kinase (BTK) inhibitor indicated for the treatment of mantle cell lymphoma (MCL). Erdafitinib (Balversa^TM^) also belongs to the family of tyrosine kinases and has been authorized for the treatment of bladder cancer. Voxelotor (Oxbryta^TM^), which contains an aldehyde function, is indicated for the treatment of sickle cell disease, which is a hemoglobin-related disorder. Voxelotor acts as a hemoglobin oxygen-affinity modulator, increasing hemoglobin levels and decreasing hemolysis indicators in patients with this condition. Entrectinib (Rozlytrek^TM^), which contains the indazole moiety, is also a tyrosine kinase inhibitor for the treatment of ROS1-positive non-small cell lung cancer and NTRK fusion-positive solid tumors.

**Figure 17:**
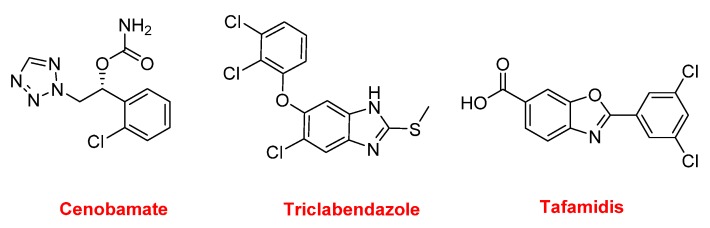
Structure of cenobamate, triclabendazole, and tafamidis.

Three other nitrogen-containing aromatic compounds were approved in 2019. Cenobamate (Xcopri^TM^) contains a tetrazole moiety and is used for the treatment of partial-onset seizures. Triclabendazole (Egaten^TM^) is a benzoimidazole and is indicated for the treatment of fascioliasis and paragonimiasis, which are liver flukes. It is also being used in veterinary medicine. Tafamidis (Vyndaqel^TM^) is a benzoxazole administered to patients with familial amyloid polyneuropathy to delay the loss of peripheral nerve function (Figure 17).

**Figure 18:**
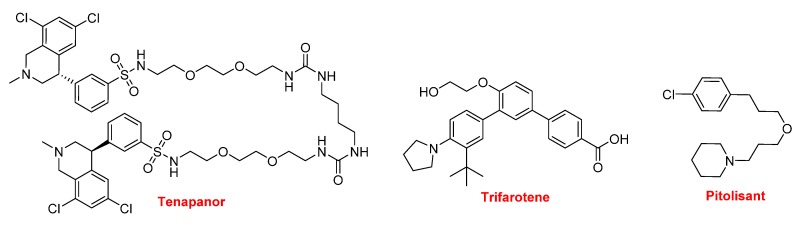
Structure of tenapanor, trifarotene, and pitolisant.

In this year, three more drugs contain nitrogen non-aromatic heterocycles were approved (Figure 18). Tenapanor (Ibsrela^TM^) inhibits the sodium-proton exchange and is used to treat irritable bowel syndrome with constipation. From a chemical structural point view, this is an interesting molecule because it is dimeric and contains dichloro-tetrahydroisoquinoline, benzenesulfonamide, and single polyethylene glycol moieties. Trifarotene (Aklief^TM^), which contains a pyrrolidine, is used for the topical treatment of acne vulgaris. Pitolisant (Wakix^TM^) is a potent and highly selective histamine 3 (H_3_) receptor antagonist. It contains a piperidine and is the first in its class. Pitolisant enhances the activity of histaminergic neurons in the brain, thereby improving wakefulness and decreasing episodes of cataplexy in those suffering from narcolepsy.

**Figure 19:**
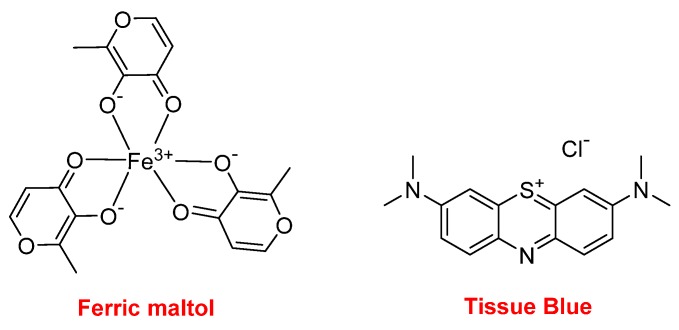
Structure of ferric maltol and Tissue Blue.

Ferric maltol (Accrufer^TM^) is an iron-chelating compound used for the treatment of patients with low iron stores (iron deficiency anemia). Also related to Fe, Tissue Blue (brilliant blue G, methylene blue, methylthioninium chloride) is a dye approved by the FDA for treating methemoglobinemia. It acts by converting the ferric iron in hemoglobin to ferrous iron (Figure 19).

Finally, this year, the FDA approved a polymer, ExEm^TM^, which is indicated as a contrast agent for imaging studies of the uterus (sonohysterosalpingography) to assess fallopian tube patency in women with known or suspected infertility. ExEm^TM^ is formed from hydroxyethyl cellulose and glycerol. 

## 3. Conclusions

**Figure 20:**
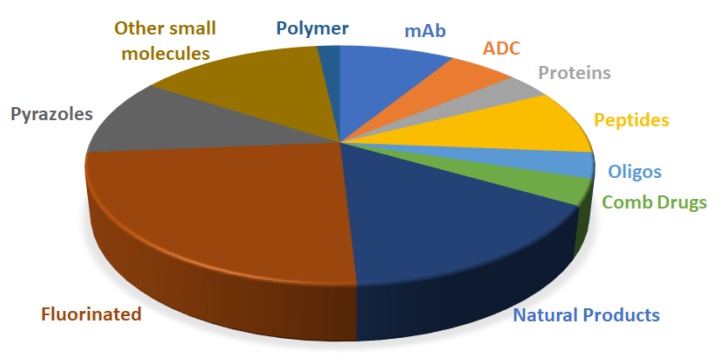
Drugs approved by the FDA in 2019 and classified on the basis of their chemical structure (drugs could belong to two different classes).

Figure 20 shows the drugs approved by the FDA in 2019 and classified on the basis of their chemical structure.

Although analysts are very cautious when addressing the pharmaceutical industry in general, and particularly regarding “Drugs to the Market”, we think that 2019 could be considered an exemplary year. It is clear that the number of the drugs accepted by the FDA this year was slightly inferior to those of 2018, which marked a record, and that there were fewer approvals of Biologics than expected. However, 2019 was marked by several milestones, thus supporting the optimistic overview of the year. Thus, the last five years (2015–2019), including the poor 2016 (with respect to the number of drugs approved), stand out as the best period in terms of FDA authorization of drugs.

We nominated patisiran (Onpattro^TM^) as the “Molecule of the Year” in 2018. This nomination was made based on the fact that patisiran was the first double-stranded siRNA drug approved, and we highlighted that it “*opens the door for the authorization of others*”. This has been confirmed this year with the approval of givosiran (Givlaari^TM^) for a different medical target. Of note, givosiran is delivered through a novel chemical-based mechanism, the so-called “enhanced stabilization chemistry (ESC)”, which again will open up new avenues for the oligonucleotide drug field. A second oligonucleotide was approved in 2019, the antisense PMO golodirsen (Vyondys 53^TM^), which follows eterlipsen (Exondys 51^TM^), another PMO authorized in 2016, thereby validating PMOs as drugs.

As discussed in the introduction, three ADCs were approved in 2019, an accomplishment considered one of the highlights of the year. Two of them, enfortumab vedotin (Padcev^TM^) and polatuzumab vedotin (Polivy^TM^) contain the peptide MMAE as payload drug, as well as a peptide base linker. Taking all this into consideration, 2019 can be deemed an excellent year for peptides. 

Of the five mAbs accepted, we would like to highlight caplacizumab (Cablivi^TM^), which is a nanobody. These chemical structures contain a single-domain antibody fragment, representing 10%–20% of the full antibody. This is the first nanobody to receive approval from the FDA. Due to the novelty and the potential for future drugs with similar characteristics, we believe that caplacizumab deserves to be nominated “Molecule of the Year 2019”.

Natural products continue to be the main inspiration for the development of new drugs. In the section of small molecules, nine APIs belong to this category. The presence of fluorine is a constant, with four of every ten small molecules containing at least one of these atoms. More important still is the concurrence of nitrogen-based aromatic heterocycles, which are present in two out of three small molecules approved this year (21 vs. 32).

It is hoped that the approval of pretomanid will bring about a new paradigm in the drug discovery process. Pretomanid was developed and registered by the TB Alliance, a non-profit organization. It took this organization 20 years to reach this point, but its achievement shows that there are alternatives to the pharmaceutical industry with respect to the development of drugs that will enhance the lives of many people. We hope that new alliances involving civil society and the public and private sectors will be built to respond to the needs of sections of society that suffer from diseases that are not of interest to the pharmaceutical industry.

As in recent years, oncology drugs received the most approvals in 2019. The novelty this year is that oncology drugs were followed by drugs indicated for neurology and hematology. Furthermore, the efforts of the pharmaceutical industry to tackle infectious diseases continues to pay off, as reflected by the drugs approved this year with these indications.

Unfortunately, the final message of this report resembles that of last year and refers to the cost of the treatments [2], which stands several of these drugs in six-digit figures. Although there is no clear solution to this challenge, we consider it pertinent to highlight this issue in these annual reports.

## Figures and Tables

**Table 1:** Biologics approved by the FDA in 2019 [1,5,6].

Active Ingredient ^a^	Trade Name ^b^	Class	Indication
Brolucizumab	Beovu^TM^	Monoclonal antibody	Wet age-related macular degeneration
Caplacizumab	Cablivi^TM^	Monoclonal antibody (Nanobody)	Thrombosis and thrombotic thrombocytopenic purpura
Crizanlizumab	Adakveo^TM^	Monoclonal antibody	Prevention of vaso-occlusive crisis in patients with Sickle cell anemia
Risankizumab	Skyrizi^TM^	Monoclonal antibody	Moderate to severe plaque psoriasis
Romosozumab	Evenity^TM^	Monoclonal antibody	Osteoporosis in postmenopausal women
Enfortumab vedotin-ejfv	Padcev^TM^	Antibody drug conjugate	Cancers expressing Nectin-4
Polatuzumab vedotin- piiq	Polivy^TM^	Antibody drug conjugate	Diffuse large B-cell lymphoma
Fam-trastuzumab deruxtecan-nxki	Enhertu^TM^	Antibody drug conjugate	Unresectable or metastatic HER2-positive breast cancer
Luspatercept	Reblozyl^TM^	Fusion Protein	Anemia in β-thalassemia and myelodysplastic syndromes
Prabotulinumtoxin (botulinum toxin)	Jeuveau^TM^	Neurotoxin protein	Temporary improvement of frown lines

^a^ By alphabetical order, ^b^ USA.
